# An Abnormal Presentation of Pediatric Genu Varum, Managed by Bilateral Tibial and Fibular Osteotomies With External Spatial Frame Placement: A Case Report

**DOI:** 10.7759/cureus.23953

**Published:** 2022-04-08

**Authors:** Robert M Chory, Ryan Cone, Susan Chory

**Affiliations:** 1 Orthopedic Surgery, Edward Via College of Osteopathic Medicine (VCOM) Auburn, Auburn, USA; 2 Obstetrics and Gynecology, Edward Via College of Osteopathic Medicine (VCOM) Auburn, Auburn, USA; 3 Education, Franklin Hospital, Floral Park, USA

**Keywords:** minimally invasive osteotomy, taylor spatial frame, tibial osteotomy, pediatric stress fracture, genu varum

## Abstract

Genu varum is a common finding in the pediatric population with a large differential, including but not limited to Blount’s disease, rickets, and physiologic bowing of the legs. Here we report a case of a 12-year-old Caucasian male who presented for an atraumatic stress fracture of the fifth metatarsal after an athletic event. Further evaluation showed significant genu varum with a Q angle of 9 degrees and medial knee joint space narrowing. The patient was unable to undergo conservative management due to early completion of puberty with relatively premature skeletal maturity. A bilateral tibial and fibular osteotomy with external spatial frame placement was performed successfully followed by six months of minor activity complicated by subclinical enoxaparin-induced purpura. The unique presentation of a stress fracture caused by compensatory mechanisms for the severe varus deformity, as well as the rarity of this procedure being performed on both legs simultaneously with good outcomes was the primary reason for the publication of this paper.

## Introduction

Genu varum, commonly referred to as bowed legs, is a common deformity of the legs causing the apex of the knee to point away from the midline. There are many pathologic causes of genu varum including achondroplasia, rickets, and Blount’s disease, but not all cases of bowed legs are pathologic in nature [1]. At birth, the normal alignment of the legs is a slight varus, and will typically increase as lower extremity weight-bearing occurs as the child begins to walk [2]. This varus alignment should resolve by adulthood and progress to a neutral or slight valgus alignment by the time orthopedic maturity is reached [2]. Risk factors for pathologic genu varum include nutritional deficiency, family history of bowed legs, and participation in soccer in adolescence [3].

Diagnosis of bowed legs should first be suspected by physical exam, and confirmed by weight-bearing lower extremity X-ray. Physical exam findings suggestive of pathologic varus deformity include lateral knee protrusion during ambulation, leg length discrepancy, and unilateral deformity [1,4]. Patient weight should also be considered as a contributing cause of lower extremity bowing, especially in obese children who have yet to reach physiologic maturity. Once the presence of a varus deformity is identified further exploration into the cause should be performed [1,4,5]. Some causes of genu varum, such as achondroplasia, are obvious on physical exam, while Blount’s disease and rickets typically require a more in-depth evaluation with imaging and lab values. The management of pathologic genu varum is complex and varies based on clinical features such as age, skeletal maturity, severity of deformity, and the underlying cause, but may involve bracing or surgical intervention [1,4,5].

## Case presentation

**Figure 1:**
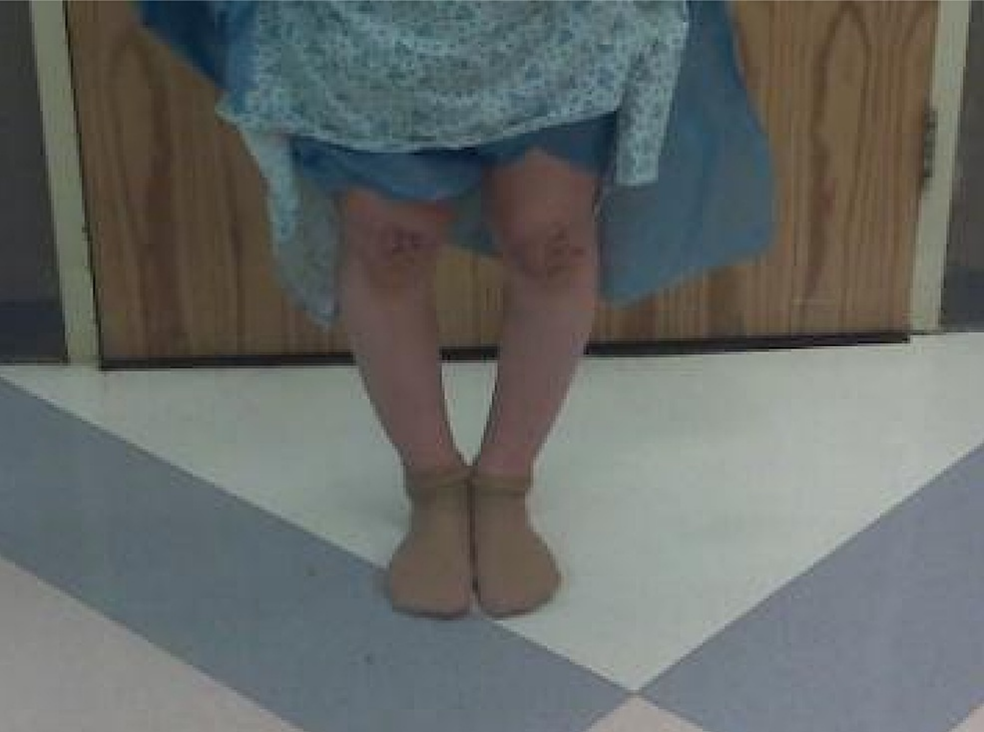
Bilateral lower extremity bowing noted on physical exam

A 12-year-old white male presented to his primary care office for evaluation of persistent right lateral foot pain that started one week prior after a soccer game. The patient denied any direct trauma to the foot other than the minor trauma associated with running. The patient was previously evaluated at birth and again at age 3 for observed varus deformity of the legs bilaterally, but treatment was deferred by their primary care physician without referral. No other significant medical history or family history was noted. On physical exam, there was minor swelling of the lateral aspect of the right foot with tenderness to palpation over the fifth metatarsal, but no major bruising, redness or other obvious deformities were noted. When asked to stand with his feet together a varus deformity of the legs bilaterally was noted, as shown in Figure 1. An X-ray of the right lower extremity was obtained, revealing a Torg 1 stress fracture of the fifth metatarsal. The patient was placed in an immobilization boot and referred to an orthopedic surgeon for further evaluation of the varus deformity.

**Figure 2:**
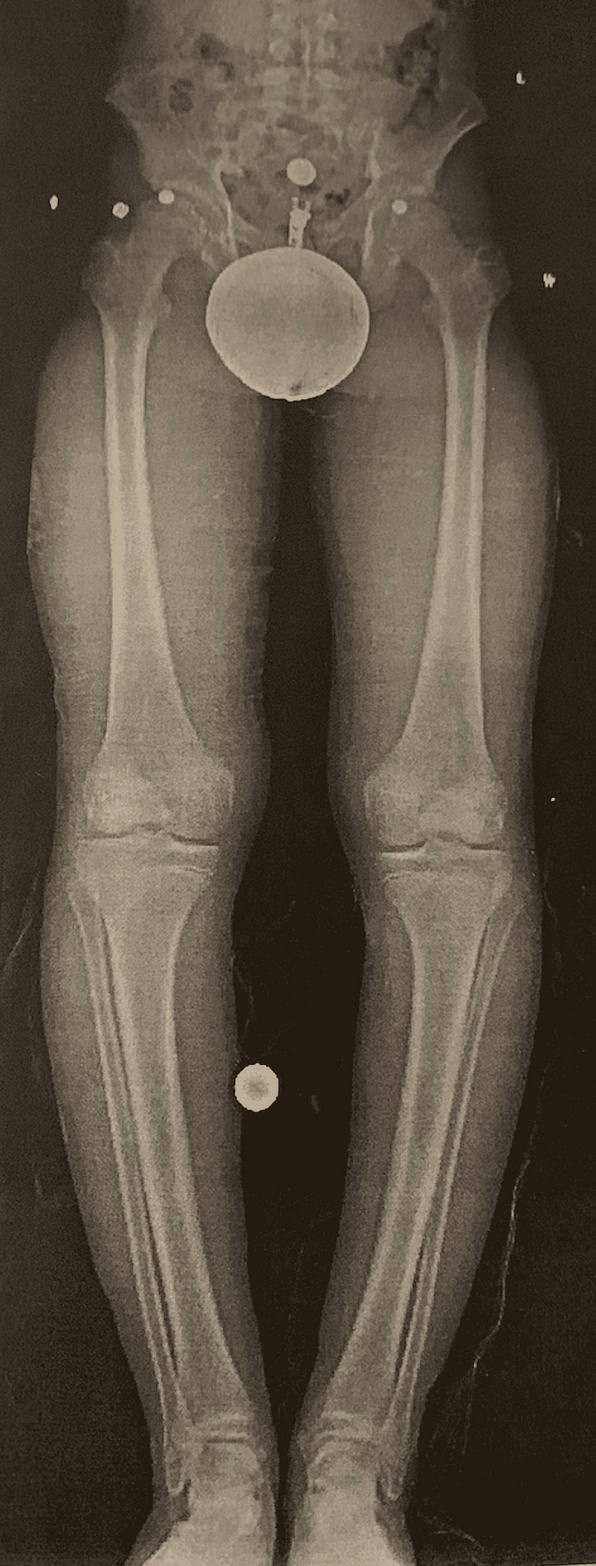
Pre-operative lower extremity X-ray showing the varus deformity bilaterally

On arrival to orthopedic surgery, a bilateral weight-bearing lower extremity X-ray was taken, demonstrating obvious tibial bowing bilaterally that was slightly worse on the right in addition to medial knee joint space narrowing bilaterally, as shown in Figure 2. A Q angle of 9 degrees was calculated using the previously mentioned lower extremity X-ray. The patient was then counseled on the cause-and-effect relationship between the bowed legs and fifth metatarsal stress fracture as well as the need for operative management to correct the lower extremity deformity and prevent future complications.

The patient underwent a bilateral high tibial osteotomy concurrently with bilateral distal fibular osteotomies and external Taylor spatial frame placement. The procedure was uncomplicated and the patient was admitted to the surgical intensive care unit for four days. Immediate physical therapy was started on post-op day 1. He was discharged home on post-op day 4 on oxycodone for pain control and enoxaparin injections. Continued ambulation was encouraged while at home within reasonable limits. Evaluation at two weeks, five weeks, nine weeks, and three months post-operation was performed to ensure proper growth progression and identify any complications. Of note, evaluation at three weeks post-op revealed purpura, attributed to enoxaparin administration. No other adverse effects were noted and labs including platelet count, complete blood count, and prothrombin time/international normalized ratio (PT/INR) were within intended limits for anticoagulation, so enoxaparin was continued with careful monitoring.

**Figure 3:**
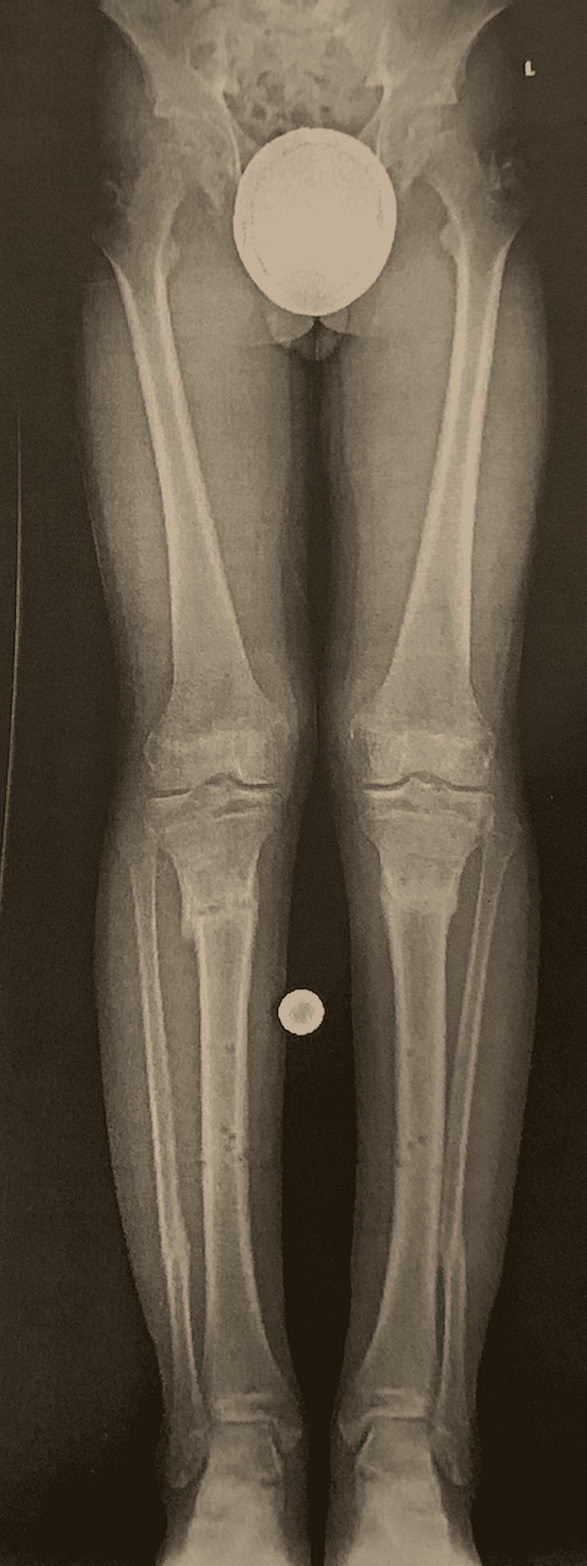
Post-operative X-ray of the lower extremity showing correction of the varus deformity

Three and a half months post-operation the patient was scheduled for removal of internal and external hardware under general anesthesia without complications. Referral to physical therapy for two months was placed and he was ultimately able to participate in full-contact sports six months after the removal of hardware. One year follow-up revealed full correction of the varus deformity, as demonstrated by the X-ray shown in Figure 3, with no abnormalities noted other than complaints of mild patella-femoral pain syndrome (PFPS). Counseling on stretches and the use of non-steroidal anti-inflammatory medications for the management of PFPS was provided. While no abnormalities were observed from an orthopedic or structural standpoint, it should be noted that the patient experienced profound psychological stress due to having a period of debilitation requiring homeschooling, with inability to participate in social and athletic events during a critical developmental time from a social standpoint.

## Discussion

The presence of genu varum in a child is most commonly due to physiologic changes associated with normal growth and can usually be managed conservatively. However, there is a small population in which bowed legs are pathologic and need further evaluation. Obese children and children who play soccer are two of the most well-described modifiable risk factors for the development or worsening of a varus deformity [3,5]. An increased incidence of genu varum has also been reported in children who have a lower intake of calcium-containing foods resulting in the development of vitamin D deficiency and subsequent development of rickets bone deformities [5].

When approaching the diagnosis of bowed legs, a holistic approach is suggested. As described in our case, management of the metatarsal stress fracture without further evaluation of the cause would have led to further delay in the management of the varus deformity, and likely other orthopedic complications [2,4]. Differentiation between the many causes of genu varum can be difficult, but the determination of pathologic versus physiologic should first be determined. Bowed legs within two standard deviations of the norm in a child under age two should be considered physiologic and monitored for worsening deformity or persistence beyond three years of age [2]. Clinical features suggestive of pathologic genu varum include but are not limited to unilateral or asymmetrical bowing, history of lower extremity fracture, and lateral thrust when walking [5]. In those who present with features of pathologic bowed legs, a bilateral, weight-bearing X-ray of the full lower extremity should be performed in addition to obtaining a vitamin D level, a serum calcium level, and referral to an orthopedic surgeon [2].

High tibial osteotomy (HTO) is one of the more commonly used surgical techniques to correct varus deformities. This procedure is typically performed unilaterally with the intent to operate on the other extremity after initial recovery of the first limb has occurred [6]. Operation on both extremities is usually a necessity due to the increased weight-bearing load placed on the non-operative limb in unilateral procedures, however, similar results have been seen when a procedure has been performed bilaterally simultaneously compared to two unilateral procedures [6].

This case describes a unique initial presentation, where a more severe varus deformity on the right resulted in a lateral stress fracture due to increased weight-bearing load and abnormal weight distribution, the same phenomenon seen when only a unilateral HTO is performed without surgical management of the opposite leg. In addition, this patient required bilateral high tibial and fibular osteotomies to be performed simultaneously to ensure equal distribution of weight-bearing load and prevent any additional complications in the post-operative period. Ultimately HTO is well tolerated, with the most common complication being loss of the correction, and is a great surgical option for the management of genu varum [7].

## Conclusions

Bowed legs are an abnormality routinely seen in the pediatric population and should be monitored for pathologic etiologies as the patient approaches adolescence. Misdiagnosis of physiologic genu varum in children can result in a delay of treatment and the need for more invasive surgical techniques later in life, as seen in this case. This patient presented with a stress fracture of the fifth metatarsal secondary to a clinically significant varus deformity bilaterally. Bilateral tibia and fibular osteotomies with external spatial frame placement were performed, and resulted in complete resolution of the deformity at one year follow-up exam. Persistent patella-femoral pain was the only noted complication and was managed conservatively with non-steroidal anti-inflammatory drugs (NSAIDs) and physical therapy/stretching. The unique initial presentation of a lateral foot fracture in addition to the rarity of bilateral tibial and fibular osteotomies being performed simultaneously, successfully, has not been well described in the literature and displays an unusual presentation and management of a common condition.
